# Supplementary material for: Folding Dynamics of 3,4,3-LI(1,2-HOPO) in Its Free and Bound State with U4+ Implicated by MD Simulations
Source: Molecules. 2022 Nov 23;27(23):8151. doi: 10.3390/molecules27238151 (PMC9740235; doi:10.3390/molecules27238151)
Supplement: Supplementary file 1 [file molecules-27-08151-s001.zip › molecules-1986720-supplementary.pdf]

Supporting Information

Folding Dynamics of 3,4,3-LI(1,2-HOPO) in Its Free and Bound State with U^{4+} Implicated by MD Simulations

Qin Wang [1,2], Ziyi Liu [2,*], Yu-Fei Song [1,*] and Dongqi Wang [2,3,*]

[1] State Key Laboratory of Chemical Resource Engineering, Beijing Advanced Innovation Center for Soft
Matter Science and Engineering, Beijing University of Chemical Technology, Beijing 100029, China; 2019400226@buct.edu.cn

[2] State Key Laboratory of Fine Chemicals, Liaoning Key Laboratory for Catalytic Conversion of Carbon
Resources, School of Chemical Engineering, Dalian University of Technology, Dalian 116024, China

[3] CAS-HKU Joint Laboratory of Metallomics on Health and Environment, Multidisciplinary Initiative Center, Institute of High Energy Physics, Chinese Academy of Sciences, Beijing 100049, China

* Correspondence: liuziyi@dlut.edu.cn (Z.L.); songyf@mail.buct.edu.cn (Y.-F.S.); wangdq@dlut.edu.cn (D.W.)

Table S1. The number of clusters sampled in the simulations of *t*-HOPO, *t*-$HOPO^{4-}$ and U^{IV}-*t*-HOPO complex systems, respectively, using three cutoff values. The proportions of main clusters in each system are also shown.

	t-HOPO			*t*-$HOPO^{4-}$			U^{IV}-*t*-HOPO complex		
Cutoff (nm)	0.05	0.18	0.35	0.05	0.18	0.35	0.05	0.18	0.35
Num of clusters	4643	244	13	2394	44	4	26	1	1
The proportion of main clusters (%)									
Cluster1	0.40	5.50	59.08	2.82	59.74	97.68	51.10	100	100
Cluster2	0.32	4.08	17.36	1.70	6.98	1.62	23.98	/	/
Cluster3	0.22	3.18	10.84	1.28	5.80	0.64	16.30	/	/
Cluster4	0.20	2.96	5.02	1.16	4.00	0.06	3.90	/	/
Cluster5	0.16	2.84	4.04	1.16	3.52	/	1.34	/	/
Cluster6	0.16	2.60	1.22	0.92	2.66	/	0.86	/	/

Table S2. The compositions of the systems to simulate *t*-HOPO, *t*-$HOPO^{4-}$ and U^{IV}-*t*-HOPO in aqueous phase.

Systems	N_{HOPO}	N_{U4+}	N_{Na}	N_{water}	Sampling time (ns)	Box size (nm^3)
t-HOPO	1	0	0	1559	100	3.844*3.678*3.391
t-$HOPO^{4-}$	1	0	4	1541	100	3.842*3.699*3.308
U^{IV}-*t*-HOPO	1	1	0	1500	100	3.459*3.602*3.683

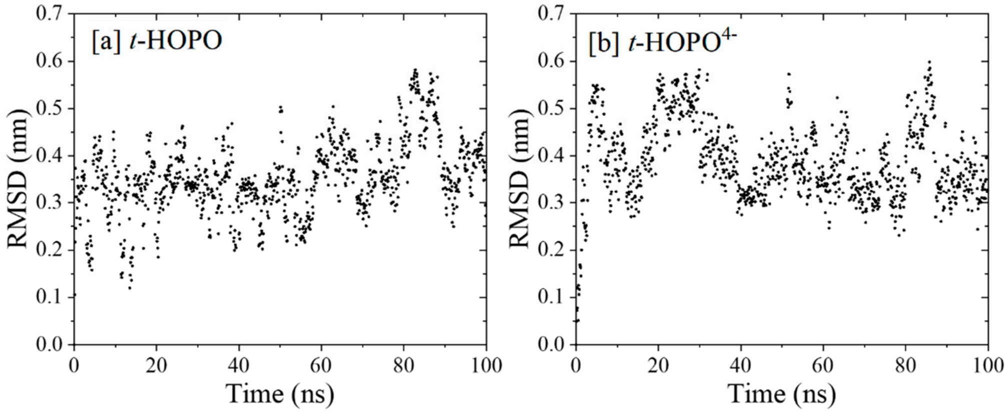

Figure S1. Atomic positional RMSD of all atoms of *t*-HOPO (a) and *t*-$HOPO^{4-}$ (b). The structure of the first frame in each simulation was used as the reference.

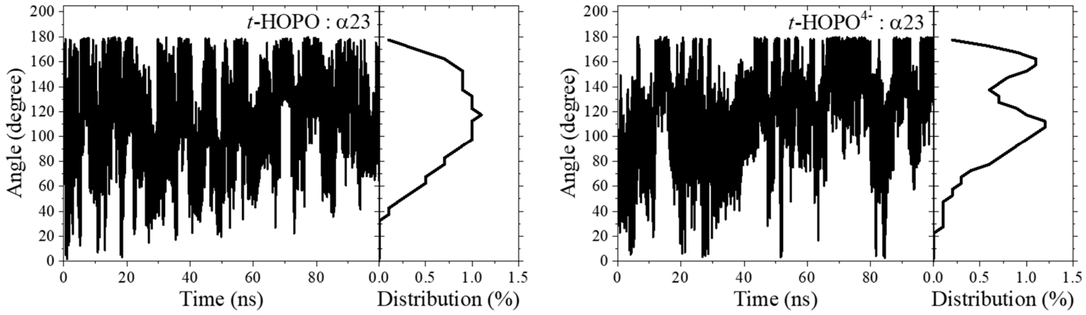

Figure S2. The time evolution of the angle between Plane2 and Plane3 (α23) and their populations in the simulations of *t*-HOPO in its neutral (left) and deprotonated (right) states.

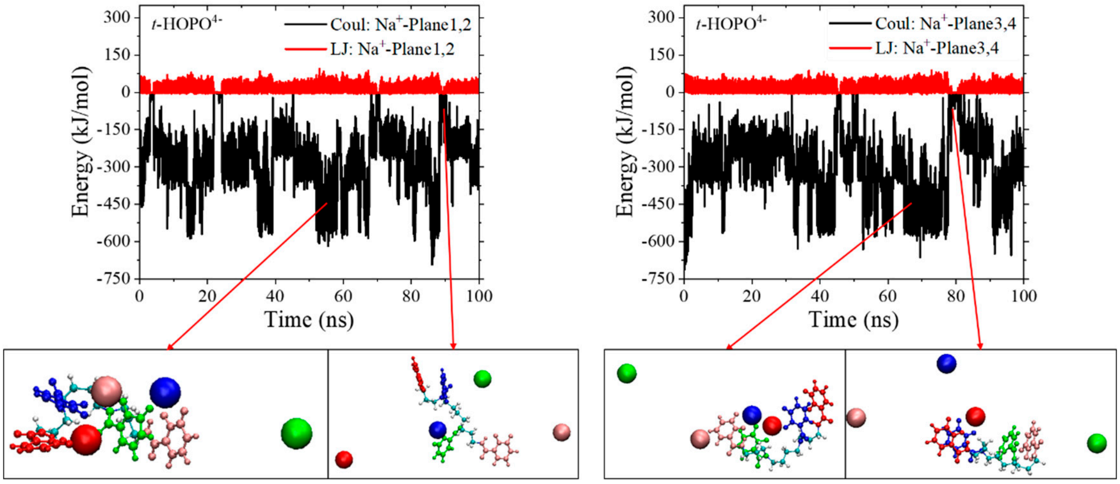

Figure S3. The time evolution of the interaction between Na^+ counterion and the m-HOPO units in the simulations of *t*-$HOPO^{4-}$ and the typical conformations in the simulation. Color scheme: Plane1 and Na1 in red, Plane2 and Na2 in blue, Plane3 and Na3 in green, Plane4 and Na4 in pink.

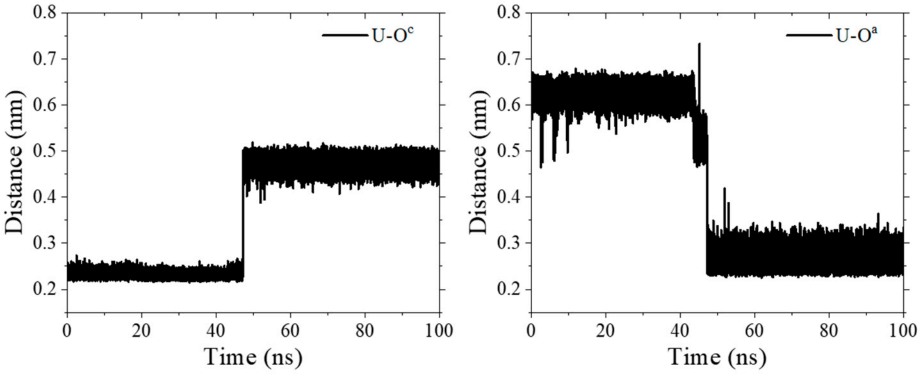

Figure S4. The time evolution of distances between U^{4+} and O^c, U^{4+} and O^a.

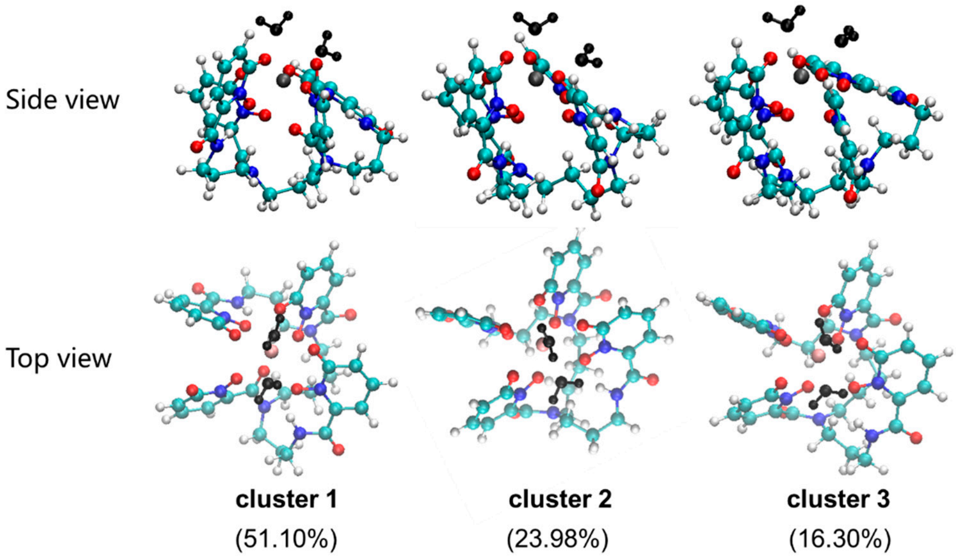

Figure S5. The central structures of the top three clusters in the simulations of U^{IV}-*t*-HOPO complex with side view (top) and top view (below). Color scheme: N atoms in blue, O atoms in red, water molecules in black, C atoms in cyan, H atoms in white, and U atom in brown.

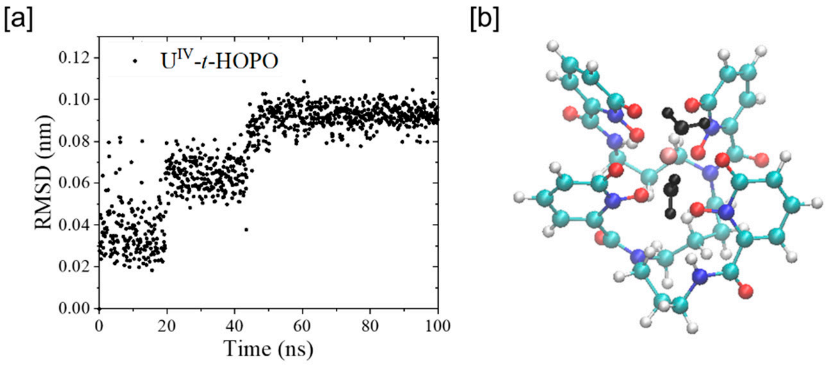

Figure S6. (a) Atomic positional RMSD of all atoms of U^{IV}-*t*-HOPO complex with the structure of first frame in the simulation used as the reference. (b) The structure of first frame in the simulation.
